# Adaptation and pilot implementation of the WHO guideline on digital intervention to strengthen mental health systems in Lagos state, Nigeria

**DOI:** 10.1186/s12961-026-01463-8

**Published:** 2026-09-10

**Authors:** Abiodun O. Adewuya, Jibril Abdulmalik, Seye Abimbola, Olabisi E. Oladipo, Asmau Dahiru, Falmata Shettima, Adedolapo Fasawe, Christine Fahim, Robert Marten, Sonam Yangchen, Sharon Straus

**Affiliations:** 1 Dept of Behavioural Medicine, Lagos State University College of Medicine, Ikeja, Lagos, Nigeria; 2 College of Medicine, University of Ibadan, Ibadan, Nigeria; 3 The University of Sydney, Sydney, Australia; 4 Centre for Mental Health Research and Initiative, Ikeja, Lagos, Nigeria; 5 Federal Neuro-Psychiatric Hospital, Maiduguri, Nigeria; 6 Lagos State Ministry of Health, Ikeja, Lagos, Nigeria; 7 Knowledge Translation Program, Li Ka Shing Knowledge Institute, St. Michael’s Hospital, 30 Bond Street, Toronto, ON M5B 1W8 Canada; 8 Alliance for Health Policy and Systems Research, 20 Avenue Appia, 1211 Geneva, Switzerland

**Keywords:** Adaptation, Pilot implementation, Digital intervention, Mental health systems, Guideline, Nigeria

## Abstract

**Background:**

The uptake and implementation of cost-effective interventions and programs for mental health problems are often threatened by weakened health systems (HS) in low- and middle-income countries (LMICs). Digital technologies provide concrete opportunities to tackle these HS challenges. This study aimed to adapt and conduct a pilot implementation of the “WHO Guideline: recommendations on digital interventions for health system strengthening” (WHO-GRDI) for mental health systems in Nigeria.

**Methods:**

The AGREE-II was used to appraise the WHO-GRDI by a team of five independent raters, and the ADAPTE framework was followed in adaptation of the WHO-GRDI to the local setting. The pilot implementation of the recommendations of the adapted WHO-GRDI was carried out within five randomly selected primary care systems. Assessment of the acceptability (using acceptability of intervention measure), appropriateness (using intervention appropriateness measure), feasibility (using feasibility of intervention measure) and perceived effectiveness of the WHO-GRDI recommendations was conducted. In addition, the stakeholders’ readiness to implement change was evaluated using the Organizational Readiness for Implementing Change scale.

**Results:**

The overall assessment of the WHO-GRDI using the AGREE-II was 79.3%. The final draft of the adapted guideline has eight recommendations. During the pilot implementation, the recommendations regarding drug stock notification, health workers’ supervision and communication and targeted client communication were considered quite effective (score range 80.0–92.0%), acceptable (score range 80.5–91.0%) and appropriate (score range 84.0–90.0%), and could be feasibly implemented (score range 81.0–90.0%) for mental health services in the local setting with a focus on mobile technology. However, the health workers expressed some concerns with recommendations on “Client-to-health-provider communication” (score range 65.6–73.4%) and “clinical decision support” (score range 78.0–79.0%).

**Conclusions:**

Our study detailed the process involved in the adaptation and pilot implementation of a newly released HS guideline for mental health services in a LMIC. There is a need for further research that will embrace the views and experiences of end-users of health systems guidelines to understand and contextualize the balance between generalizability and local adaptability of health systems guidelines in LMICs.

## Background

The most recent estimate showed that mental disorders remained amongst the top ten leading causes of burden worldwide, with the global number of disability-adjusted life-years (DALYS) due to mental disorders at 125.3 million. More than 4.9% of DALYs and 14.6% of years lived with disability (YLDs) are attributable to mental disorders [1]. Nearly 20% of this burden is from low- and middle-income countries (LMICs) [1]. When untreated, MH problems lead to severe disability, causing enormous social and financial burden on families and society. Lagos State is one of the 36 states in Nigeria and accounts for 20 million of the 200 million people in Nigeria [2]. The recent Lagos State Mental Health Survey revealed a high level of common MH disorders and suicidal risk amongst the citizens of Lagos [3, 4]. In Nigeria and other LMICs, despite the huge burden of MH problems, up to 85% of people with mental disorders do not receive any form of treatment [5, 6].

To reduce this huge MH “treatment gap”, the WHO recently introduced the Mental Health Gap Action Programme (mhGAP) to guide the integration of MH services and interventions into primary care in LMICs [7, 8]. The mhGAP intervention guide (mhGAP-IG) had been contextualized and adapted for the Nigerian setting [9]. The Mental Health in Primary Care (MeHPriC) program in Lagos State, Nigeria, was based on the mhGAP [10]. The components of the MeHPriC program include (a) diagnosing and management of common mental and neurological disorders (such as depression, anxiety, substance use disorders and epilepsy); (b) mental health promotion; (c) offering mental health first aid and providing counselling and psychosocial supports to people in distress; and (d) referral pathway for emergency cases. The MeHPriC program had shown a high level of acceptability and considerable clinical and cost-effectiveness and thus became adopted and operational in the 57 flagship PHCs in Lagos, Nigeria. However, it has been noted that the uptake and implementation of such lifesaving and cost-effective interventions and programs such as the mhGAP are often threatened by weakened health systems, significant resource limitations and numerous competing health and other priorities in these LMICs [11]. Geographical inaccessibility, low demand for services, delayed provision of care, and low adherence to clinical protocols limit the ability to close the gaps in coverage, quality and affordability of interventions and undermine the potential to achieve success [12].

Increasingly, it is being recognized that digital technologies provide concrete opportunities to tackle health system challenges (especially those related to service delivery and human resources), and thereby offer the potential to enhance the coverage and quality of health practices and services, especially in LMICs with scarce human resources, huge hard-to-reach populations and several conflict-inflicted regions [13]. The explosive growth and deep penetration of mobile communications provide millions of rural dwellers access to reliable technology for communication and data transfer. The handiness and widespread adoption of this technology and people’s attachment to their mobile phones made it an attractive avenue for delivering health interventions [14].

To guide the process of strengthening health systems for member countries, the WHO developed evidence-based health systems guidelines to enhance coverage, quality, efficiency, and equity. The “WHO Guideline: recommendations on digital interventions for health system strengthening” (WHO-GRDI) [15] provides evidence-based recommendations on digital health interventions as they affect clients, health workers, health system managers and data services. The WHO-GRDI includes nine recommendations on birth notification, death notification, stock notification and commodity management, client-to-provider telemedicine, provider-to-provider telemedicine, targeted client communication, tracking of patients’/clients’ health status and service, health worker decision support, and provision of training and educational content to health workers.

Understanding and contextualizing the generalizability and local adaptability of health guidelines are quite important and may improve local uptake. Guideline adaptation is an effective alternative to de novo guideline development, especially in LMICs. This is to avoid duplication of effort, use available resources cost-effectively and facilitate customization of the guidelines for local health system settings. Since the WHO-GRDI was recently released and not specifically designed for mental health services, there is an urgent need to adapt and pilot test the implementation of WHO-GRDI for mental health systems, especially in LMICs. Guideline adaptation requires a process that is active, systematic and participatory and preserves the integrity of the original guideline. This paper describes the process involved in the adaptation and pilot implementation of the WHO-GRDI for mental health systems in Lagos State, Nigeria. We specifically aimed to identify the challenges to implementing the adapted WHO-GRDI for mental health service in Lagos and also identify possible strategies to overcome such challenges.

## Methods

### Study design

The project was based on the Knowledge to Action cycle, [16] which offers a model for promoting the use of guidelines. It has two concepts: “knowledge creation”, which includes knowledge inquiry, synthesis and tools, and “action cycle”, which represents the activities that will be needed for knowledge application and include (1) identifying the problem that needs addressing; (2) selecting the knowledge relevant to the problem; (3) adapting the identified knowledge to the local context; (4) assessing barriers to using the knowledge; (5) selecting, tailoring and implementing interventions to promote the use of knowledge; (6) monitoring knowledge use; (7) evaluating the outcomes of using the knowledge; and (8) sustaining ongoing knowledge use.

### Setting

The project was conducted in Lagos State, Nigeria. Lagos State, with 57 Local Council Development Authorities (LCDA), has about 200 primary care facilities. Out of the 3–4 PHCs in each of the LCDAs, one is designated a “flagship PHC” with the full complement of doctors, nurses, midwives, community health extension workers, pharmacy technicians and counsellors. The “flagship PHC” takes referrals from the other PHCs in the LCDA. Lagos State has made attempts to strengthen MH systems, especially in the areas of governance (by setting up the MH desk office in the state ministry of health and launching the Lagos State Mental Health Policy), finance (by ensuring that there is a distinct budget for MH in the state), pharmaceuticals (by ensuring the availability of essential psychotropic drugs at public health facilities), human resources (by adopting “task-shifting” policy for MH services) and service delivery (incorporating mental health in primary care via the MeHPriC project). The MeHPriC project runs in each of the 57 “flagship PHCs” in the state.

### Ethics approvals

Ethics approvals were obtained from Ethics Committees of both the Lagos State University Teaching Hospital (LREC/06/10/1294) and the World Health Organization (ERC.0003300).

### Phases of the project

**Fig. 1:**
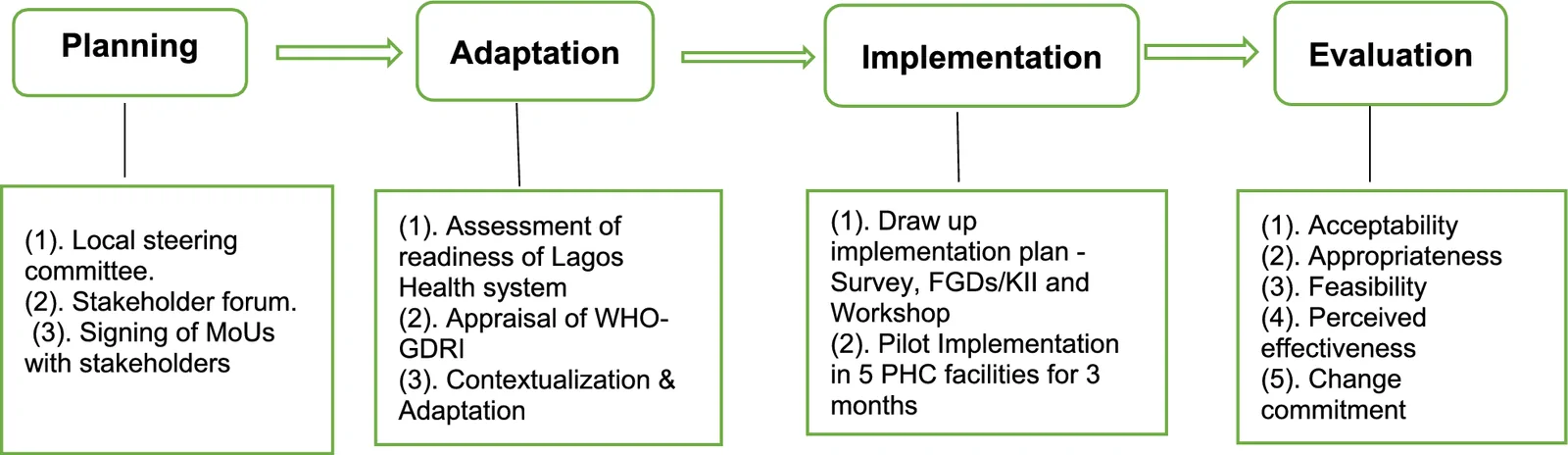
Flowchart of the project

The phases of the project are shown in Fig. 1, and include the planning phase, adaptation phase, implementation phase and evaluation phase.

1. Planning phase: To facilitate successful implementation and cooperation of the health workers and other stakeholders, we disseminated project information early, contacting existing networks such as unions of nurses, midwives, community health workers and general practitioners in Lagos State, as well as the state health ministry. We followed the ADAPTE framework. The ADAPTE process [17] provides a systematic approach to adapting guidelines produced in one setting for use in a different cultural and organizational context. It ensures that the adapted guideline not only addresses specific health questions relevant to the context of use, but is also suited to the needs, priorities, legislation, policies and resources in the targeted setting. We first established a Local Steering Committee (LSC) consisting of members of the project team and four external members including identified local experts in digital intervention, health guideline development, health program implementation, and qualitative interviews. The LSC was tasked with overall project supervision, monitoring, establishing quality and standards and providing advice on scientific credibility and contextual factors in the local environment. We also established the Stakeholders’ Forum (SF), consisting of two representatives each of the local mental healthcare providers (one psychiatrist and one psychologist), primary healthcare (PHC) workers (one nurse and one doctor), digital technology providers and policymakers and nongovernmental organizations. We recruited highly respected and influential members of the community after ascertaining their availability, disposition, willingness to participate in the project and non-declaration of any conflict of interest. The members of the SF were not part of the LSC, and the SF’s role was to provide support and advice to the LSC for the successful implementation of the project and facilitate adoption. The LSC developed and adopted a set of guiding principles for the adaptation and pilot implementation process. We formalized the adaptation and pilot implementation plans with timelines.
2. Adaptation phase: In this phase, we conducted the following:
  - Assessment of the readiness of the Lagos health system for the use of WHO-GRDI: We did this in two ways: (1) we identified, retrieved and reviewed the existing digital laws, policies and current regulations and practices regarding digital intervention and mental health in Nigeria. We also reviewed the current state of implementation of the MeHPriC Lagos project, including examination of the project toolkits and manuals (for intervention, monitoring/supervision and training) to develop an understanding of how digital interventions can be incorporated into the context, process and progress of the MeHPriC project. Two independent reviewers using a designed critical-appraisal checklist systematically performed a content and quality appraisal of each document and extracted all information necessary to guide the use of digital intervention for health for mental health systems in Nigeria. Summaries of the extracted data were provided as narratives and the possible implications on the planned implementation of digital intervention for mental health systems in Nigeria were outlined. (2) We conducted a mapping of already existing digital interventions in Nigeria using the Digital Health Atlas (DHA) [18, 19]. The DHA is an open-source web platform designed to support governments, technologists, implementers and donors to better coordinate digital health activities globally. Two independent reviewers mapped Nigeria and other sub-Saharan African digital health investment functions and health focus. They then reviewed the existing interventions for geographic scope, digital health implementation, health system challenges, technology overview and interoperability and standards. Summaries were provided with essential components outlined.
  - Determination and clarification of the health question: We prepared a Population, Intervention, Professionals, Outcomes, and Health care settings (PIPOH) summary for the project.
  - Appraising the WHO-GRDI: We appraised the WHO-GRDI using the second edition of the Appraisal of Guidelines for Research and Evaluation (AGREE-II) Instrument [20]. The AGREE-II was developed to (a) provide a framework to assess the quality of guidelines, (b) provide a methodological strategy for the development of guidelines and (c) describe what information and how the information ought to be reported in guidelines. The AGREE-II consists of 23 key items organized within 6 domains (scope and purpose, stakeholders’ involvement, rigour of development, clarity of presentation, applicability and editorial independence) followed by two global rating items. Each of the AGREE-II items and the two global rating items are rated on a 7-point scale (1: strongly disagree to 7: strongly agree). The team (*n* = 5) underwent a 5-day online training before the exercise. Each member of the team independently scored the WHO-GRDI using the AGREE-II scale.
  - Contextualization and adaptation: An 11-member Contextualization and Adaptation Panel was set up that included mental health specialists, primary care specialists, policymakers, digital, digital health technology providers and local linguists. We recruited the members on the basis of a detailed stakeholder analysis and identification process, ensuring that only local actors who are familiar with the terrain and local health system were recruited from local research and academic institutions, healthcare facilities, relevant private corporations and government ministries. Each member was provided with the WHO-GRDI. A research team member first presented the results from the assessment of the readiness of the Lagos health systems and the appraisal of the WHO-GRDI to the panel and briefed the members on the basis of the guideline. Feedback was obtained about the structure and content, ease of use, readability and general acceptability of the WHO-GRDI, possible modifications and alignment with the Nigerian health system context. Both open-ended and structured feedback was provided on the overall strengths and weaknesses of the WHO-GRDI recommendations. Multiple rounds of feedback were gathered, which were outlined, and a formal consensus method was used to arrive at the adaptations to be made to the WHO-GRDI. The panel then performed a preliminary content adaptation of the WHO-GRDI. A draft document was produced, which was sent to the local professional bodies for external review.
  - Finalization: A series of consultation meetings were conducted with local stakeholders. After conducting a stakeholder analysis and identification process, we purposely recruited a total of 60 participants (10 mental health workers, 10 primary care workers, 10 policymakers, 20 mental health service users, 5 digital health technology providers and 5 health service researchers) from local research and academic institutions, healthcare facilities, relevant private corporations and government ministries. Via email, the participants were sent electronic copies of the adapted WHO-GRDI and a short online survey questionnaire asking about whether they agree with or approve the draft, their views on the strengths and weaknesses of the draft, suggested modifications, confidence in the adaptation process, whether they would use the guideline and how the WHO-GRDI recommendations would impact the mental health systems in Nigeria. Endorsement by the primary healthcare workers’ associations was sought.
3. Implementation phase: This phase consisted of drawing up the implementation plan (through surveys, interviews and a workshop), the pilot implementation and the evaluation of the implementation.
  - Drawing up implementation plan: This involved three activities. (a) First, participants in the adaptation stage who were interested in further participating in the project were asked to indicate their interest for possible selection. A total of 20 participants from the adaptation stage were recruited after reviewing their profiles. The consented stakeholders were sent the final copies of the WHO-GRDI with survey questions. Apart from the demographics, professional roles and responsibilities, the survey inquired about perceived digital intervention guideline priorities and factors affecting their uptake and adoption. In developing the survey, the Theoretical Domains Framework (TDF) was used to identify the possible barriers and facilitators that must be addressed for the successful implementation of the WHO-GDRI. The TDF [21] consists of 14 domains. We asked the respondents their views on a list of factors generated as possible barriers to the use of WHO digital intervention guidelines for mental health services in the state. We also asked the participants to state their views on the prioritized recommendations (using AGREE-II) contained in the WHO-GRDI for implementation in scaling up mental health services. (b) After analysis of the survey report, we then conducted 6 focus group discussions (FGDs) and 10 key informant interviews (KIIs) amongst the stakeholders earlier identified in the adaptation stage with the objectives of identifying and exploring priorities, barriers and facilitators to the implementation of the WHO-GRDI recommendations at the levels of healthcare recipient/citizen, health professional, organization and system. The FGDs (of about 5–8 per group) were organized by cadre to mitigate power imbalances and facilitate disclosure by participants. The KIIs were conducted with five service users and five mental healthcare professionals identified via the stakeholders’ analysis. (c) Finally, we conducted a series (*n* = 3) of 2-day face-to-face workshops aiming to collate multiple perspectives (from the survey, FGDs and KIIs) to select priority recommendations of the WHO-GRDI for implementation, identify potential implementation strategies to improve guideline uptake and sustainability and develop pilot implementation and evaluation plans. The workshop participants (*n* = 36) were purposefully sampled ensuring representation from across the healthcare system, healthcare administrators, policymakers, nongovernmental organization staff, representatives from professional associations, frontline healthcare providers and healthcare system researchers/academics and service users’ groups. Using an electronic audience response system, workshop participants completed an anonymous individual ranking exercise on the basis of the modified Delphi technique, with participants ranking priority recommendations in terms of relevance, adaptability and feasibility for implementation in the local context.
  - Pilot implementation: Through a stratified random sampling procedure, one PHC facility involved in the MeHPriC project was randomly selected from each of the five administrative districts in Lagos. All the selected PHC facilities consented to participate in the study. In each of the participating PHC facilities, trained health workers (*n* = 11) implemented the adapted WHO-GRDI for 3 months.
4. Evaluation Phase: Trained evaluators (*n* = 10) collected data on the pilot implementation outcomes, which included acceptability (using acceptability of intervention measure; AIM), appropriateness (using intervention appropriateness measure; IAM) and feasibility (using feasibility of intervention measure; FIM). Each of the AIM, IAM and FIM [22] is a 4-item instrument measured on a 5-point Likert scale that ranged from “completely disagree” (score 1) to “completely agree” (score 5). Scores range from 4 to 20, with higher scores indicating greater acceptability, appropriateness or feasibility. Perceived effectiveness was assessed with a 1-item scale asking the participants about their perception of the effectiveness of the intervention with scores ranging from “not effective” (score 1) to “very effective” (score 5). Change commitment and efficacy was measured with Organizational Readiness for Implementing Change (ORIC) scale [23]. The 12-item ORIC scale consists of 5 items reflecting organizational members’ shared resolve to implement a change (change commitment) and 7 items reflecting organizational members’ shared belief in their collective capacity to implement a change (change efficacy). Each of these 12 items is scored using a 5-point Likert scale ranging from “disagree” (1) to “agree” (5) with a score range of 12–60 and higher scores reflecting higher readiness to change. The evaluation was conducted with clients (*n* = 150), health workers (*n* = 50), a mental health team (*n* = 5) and health managers (*n* = 5). Whilst all the participants completed the AIM, IAM, FIM and perceived effectiveness scale, only the health workers and health managers completed the ORIC.

### Analysis

The survey data were analysed as descriptive statistics. For ease of interpretation, the mean scores on AIM, IAM, FIM and perceived effectiveness scale were converted to percentage scores. A cutoff score of 16 and above (80%) on AIM, IAM and FIM and cutoff score of 4 and above (80%) on the effectiveness rating was used to signify a high level of acceptability, appropriateness, feasibility and effectiveness, respectively. The mean scores on the ORIC were compared between the health workers and health managers. Following participants’ consent, we audio-taped and transcribed all the FGDs, KIIs and workshop group discussions. Two independent analysts analysed the transcripts and field notes separately using a thematic content analysis approach [24]. All transcripts were coded by the analysts independently using the designed framework. Interrater reliability between the two analysts was performed using percentage agreement with 75% considered acceptable. Disagreements were resolved with consensus. Analyses were completed using NVivo 10 software [25].

## Results

### Adaptation stage

#### Set-up

The LSC met once a month to review the project’s progress and submitted their reports to the SF, which met quarterly to consider the project’s progress and reports and made recommendations to the LSC.

#### Readiness of the Lagos health systems

The mental health documents reviewed include the Lagos State Mental Health Law 2019 [26], Nigeria Mental Health Policy [27], Nigeria Health Task Shifting Policy [28] and the Nigeria Report on the WHO—Assessment Instrument for Mental Health Systems [29]. The digital health intervention documents reviewed include the Nigeria National Health ICT Strategic Framework 2015–2020 [30] and the Guidelines on Monitoring and Evaluating Digital Health Interventions [31]. The review showed that the health laws and policies in Nigeria encourage the use of digital and mobile technology to support health interventions especially in the areas of workers training (for task shifting) and health information dissemination to clients. There is also an existing digital health framework [30]. As of August 2021, Nigeria has more than 188 million active Global System for Mobile Communications (GSM) lines, and more than 140 million access internet services over their phones [32]. The broadband penetration in Nigeria was 48% with 78 million subscriptions [33]. The quality of service was also found to be adequate with a call setup success rate of 98.9% [34]. The MeHPriC project provides mental health services at the 57 PHCs that were designated flagship PHCs out of the nearly 200 PHCs in the state. Although most of the processes are handled manually, the MeHPriC project was using Short Message Service (SMS) and WhatsApp messages to support adherence to medications and clinic appointments and also has emergency telephone lines for acute psychological distress. Using the DHA and the Nigerian Digital Health Dashboard, 12 projects (6 national and 6 subnational) were reviewed from Nigeria, with 40% focussed on clinical interventions, 30% on surveillance and disease monitoring and 30% on health insurance and payment. None of the reviewed projects were focussed on mental health. The digital health intervention was selected after the LSC reviewed the Classification of Digital Health Interventions [35], and the PIPOH summary is detailed in Table 1.

**Table 1:** PIPOH summary

1	Population	(1) Clients with mental health problems and their families, (2) mental health and primary healthcare workers and (3) policymakers
2	Interventions	Digital mobile health interventions target clients, healthcare providers and health system managers
3	Professionals	Healthcare providers, health system managers and other stakeholders in digital health interventions
4	Expected outcomes	The adaptation of pilot implementation of WHO-GRDI for mental health systems in Nigeria in a way that is acceptable and feasible within the local environment and using the limited available resources
5	Healthcare setting	The mental health system of Lagos State, Nigeria

#### Appraisal of the WHO-GRDI

The average score for domain 1 (scope and purpose) was 73.6%, domain 2 (stakeholders’ involvement) 77.8%, domain 3 (rigour of development) 74.1%, domain 4 (clarity of presentation) 75.3%, domain 5 (applicability) 71.5% and domain 6 (editorial independence) 84.6%. The overall assessment was rated 79.3%. It was noted that the WHO-GRDI was published in 2019. The content was judged to be adequate, and there was a high level of consistency between the interpretation of evidence and recommendations of the WHO-GRDI.

#### Production of the draft adapted guideline

The Contextualization and Adaptation Panel met over 7 days (two face-to-face and four online meetings). Generally, most of the recommendations in the WHO-GRDI were accepted, with a few amendments made to the wording to align with the local setting and make it simpler for the end-users to comprehend. For the external review, the draft adapted guideline was sent to local end-users and professional bodies (including Primary Care Physicians Association and Medical Guild) for feedback. A total of six consultation meetings were held with these local stakeholders with subsequent minor modifications made to the wording. A final draft, which was agreed upon by the external reviewers, was then produced. The final draft of the adapted guideline has eight recommendations, as the “Birth notification” and “Death notification” in the original WHO-GRDI were collapsed as a single recommendation to eliminate duplication of functions as the mode, method and personnel involved with recoding live deliveries, still births and deaths in the health facilities all over Lagos state are the same. In addition, the word “telemedicine” was removed, as it was agreed that it has ambiguous meaning to workers and only mobile devices could be used in the setting for now. “Health workers’ decision support” was also changed to “Clinical decision support”. The modifications made are outlined in Table 2.

**Table 2:** Adaptations made to WHO-GRDI Recommendations

	Original WHO-GRDI recommendations	Adapted WHO-GRDI recommendations
1	Birth notification	Birth and death notification
2	Death notification	
3	Stock notification and commodity management	Drug stock notification
4	Client-to-provider telemedicine	Client-to-health-provider communication
5	Provider-to-provider telemedicine	Health workers’ supervision and communication
6	Targeted client communication	Targeted client communication
7	Health worker decision support	Clinical decision support
8	Digital tracking of patients’ health status and services within a health record	Digital health records
9	Provision of educational and training content to health workers	Digital health workers training

#### Implementation plan

The workshop identified 11 health systems challenges affecting the MeHPriC program at the level of clients, health workers and the policymakers/health managers that are modifiable using the WHO-GRDI recommendations (Table 3). At the workshop, stakeholders agreed that only five of the WHO-GRDI recommendations could be feasibly implemented within the framework and timeline of the pilot project. This included drug stock notification, client-to-health-provider communication, health workers’ supervision and communication, targeted client communication and clinical decision support. The reasons adduced for excluding the other three recommendations (birth/death notification, digital health records and digital health workers training) include non-availability of supporting infrastructure and personnel, non-peculiarity to mental health services and stakeholders not considering them of top priority for pilot implementation.

**Table 3:** Health system challenges of the MeHPriC program proposed digital health interventions as recommended by WHO-GRDI

	HS challenge	Current intervention in MeHPriC	Digital health intervention proposed	Adapted WHO-GRDI recommendation	End users
Client related					
1	Lack of information about symptoms of MH problems	Radio and TV	SMS messages on MH	Targeted client communication	Clients with MH problems
2	Lack of information about health facilities for MH problems	Radio and TV	SMS messages	Targeted client communication	Clients with MH problems
3	Nonadherence to medications and clinics appointments	SMS messages	SMS and WhatsApp messages	Targeted client communication	Clients with MH problems
4	Emergency help for acute distress	Telephone helpline	Telephone helpline; SMS and WhatsApp alerts	Client-to-health-provider communication	Clients with MH problems
Health worker related					
5	Problem with appropriate diagnosis and treatment of MH problems by health workers	MeHPriC diagnosis and intervention manual	e-Versions of MeHPriC manual and mhGAP-IG on palmtops and mobile phones	Clinical decision support	Health workers
6	Sustaining clinical competence and adherence to intervention guidelines	Physical visit to the facility by MH specialist	Clinical supervision using video/WhatsApp	Health workers’ supervision and communication	Health workers
7	Continuous medical education on MH	Yearly 2-day workshop	Digital seminars and CMEs	Digital health workers training	Health workers
8	Inadequate supply of psychotropic medications	Manual request and ledger	Digital stock monitoring across facilities	Drug stock notification	Health workers
9	Health status and evidence of impact of intervention	Manual collection of data	Digital patients health status report	Digital health records	Health workers
10	Research and epidemiology	Manual collection of data	Digital epidemiological data gathering	Birth and death notification	Health workers
Policymaker related					
11	Investment/Effectiveness of interventions	Manual collection of data	Digital patients health status report	Digital health records	Policymakers and health managers

#### Pilot implementation

The description of digital interventions available at the five selected PHC facilities is presented in Table 4. In each PHC facility, 11 health workers (2 PHC doctors, 5 PHC nurses/midwives, 2 community health officers and 2 pharmacy technicians) who were already part of the MeHPriC project were provided with either mobile phones or palmtops/tablets already preloaded with digital information for the project. Training of the 55 health workers and the 5 members of the supervising MH specialist team in the use of the digital applications took place over 2 weeks.

**Table 4:** Pilot implementation program in the 5 PHC facilities

WHO-GRDI Recommendation	Description of digital interventions available
Drug stock notification	• SMS/WhatsApp and Digital Dashboard to manage and monitor supply of psychotropic medications, which include: 1. Track inventories and supply of medications within the PHC facility and other PHC facilities in the MeHPriC project 2. Notify stock level of psychotropic medications for PHC facilities within the MeHPriC project 3. Forecast demand and manage distribution of psychotropic medications • Two pharmacy technicians per PHC facility trained to use the applications
Client-to-health-provider communication	• The 5 PHC facilities provided with dedicated 24-h helplines for calls and SMS and WhatsApp messages that clients could communicate with for psychological emergency/acute distress cases • 5 health workers per PHC facility were trained to provide psychological counselling in emergency situations
Health workers’ supervision and communication	• The monthly supervisory visits by the MH specialist done via digital platform (WhatsApp call or Zoom) • Free flow digital exchange of information between MH specialist and the PHC workers to enhance decision-making and referrals
Targeted client communication	• 50 randomly selected clients per PHC facility get WhatsApp and SMS messages on mental health event alerts, monthly clinic and medication reminders, relapse symptoms information and health promotion information
Clinical decision support	• Health workers provided with the e-version of both the mhGAP-IG and the MeHPriC intervention toolkits on their palmtops and mobile phones

#### Evaluation of pilot implementation

Table 5 presents the mean scores (and appropriate percentage score) for each of the five WHO-GRDI recommendations on AIM (acceptability), IAM (appropriateness), FIM (feasibility) and the effectiveness scale as rated by the health workers, clients and health managers. With a score of 80% (mean score 16 on AIM, IAM and FIM and mean score 4 on effectiveness rating) and above categorized as acceptable, the table presented that recommendations regarding drug stock notification, health workers’ supervision and communication and targeted client communication were considered quite effective (range 80.0–92.0%), acceptable (range 80.5–91.0%), appropriate (range 84.0–90.0%) and feasible (range 81.0–90.0%). However, the health workers notably gave low scores on all the parameters for the recommendation on “Client-to-health-provider communication” (score range 65.6–73.4%) and on acceptability and perceived effectiveness on “Clinical decision support” (score range 78.0–79.0%). The ORIC scale was completed by 32 health workers (64%) and 5 health managers (100%), and the total mean score was 55.62 (SD 3.45). There was no significant difference between the scores by health workers (mean 55.68, SD 3.45) and health managers (mean 55.20, SD 2.83).

**Table 5:** Evaluation of pilot implementation of the digital interventions

Digital intervention	Evaluators	Sample size	Acceptability (AIM) Mean and % score	Appropriateness (IAM) Mean and % score	Feasibility (FIM) Mean and % score	Perceived effectiveness Mean and % score
Drug stock notification	Pharmacy technicians	10	16.10 (80.5%)	18.00 (90.0%)	17.21 (86.1%)	4.00 (80.0%)
	Health managers	5	16.82 (84.1%)	17.23 (86.2%)	18.00 (90.0%)	4.60 (92.0%)
Client-to-health-provider communication	Health workers	25	**14.40 (72.0%)**	**13.24 (66.2%)**	**14.60 (73.0%)**	**3.28 (65.6%)**
	Clients	50	17.21(86.0%)	18.24 (91.2%)	16.70 (83.5%)	4.20 (84.0%)
	Health managers	5	16.25 (81.2%)	17.40 (87.0%)	16.30 (81.5%)	4.00 (80.0%)
Health workers’ supervision and communication	Health workers	42	16.80 (84.0%)	17.22 (86.1%)	17.51 (87.6%)	4.10 (82.0%)
	MH team	5	18.20 (91.0%)	17.39 (86.9%)	16.90 (84.5%)	4.60 (92.0%)
	Health managers	5	16.20 (81.0%)	16.80 (84.0%)	16.35 (81.7%)	4.10 (82.0%)
Clinical decision support	Health workers	42	**15.80 (79.0%)**	16.30 (81.5%)	17.30 (86.5%)	**3.90 (78.0%)**
	MH team	5	17.30 (86.5%)	17.52 (87.6%)	18.20 (91.0%)	4.10 (82.0%)
	Health managers	5	16.50 (82.5%)	17.10 (85.5%)	17.82 (89.1%)	4.10 (82.0%)
Targeted client communication	Clients	146	18.20 (91.0%)	17.32 (86.6%)	17.30 (86.5%)	4.30 (86.0%)

The figures in bold are those below the 80% score considered “acceptable”

The summary of the qualitative assessments (Table 6) showed that a large proportion of the health workers believed that a majority of the recommendations of the WHO-GRDI will improve and ease MH intervention and the health workers’ ability to relate well with MH specialists and provide flexible intervention options. The health workers, however, had concerns regarding the poor electricity and network connectivity, the need for more training in using the digital devices and the safety of the mobile devices.

**Table 6:** Health workers’ views on opportunities and challenges of implementing the WHO-GRDI

	Opportunities of WHO-GRDI	Challenges of WHO-GRDI
1	Will make MH interventions fast and easier	May not be feasible where electricity and network connectivity are poor
2	Will improve ability of health workers to relate well with other professionals and MH specialists	More training needed to use and be familiar with the digital platforms
3	Will allow health workers to expand their range of interventions and give flexible options	Mobile devices may be lost or stolen
	Quotes on perceived challenges of “Client to health providers communication” recommendation	
	HW 1: “Although it is good for the patients to be able to communicate easily and fast with the health workers, but I feel this may be dangerous especially in patients who maybe acutely disturbed and probably considering suicide…..The case will definitely warrant a face to face meeting, I am not sure a digital platform can help with that….also if unfortunately the patient end up committing suicide, I will feel guilty that I could have helped better if it were to be a face to face conversation where I could assess the suicidal risk better….” HW 2: “The case of client to health recommendations is somehow risky to me. …if I don’t have a full picture of the case, how would I be able to make diagnosis and commence appropriate management…..and let me ask, would I be held liable if something happened to the patient?….I am not sure that will be fair…” HW 3: “…So does that mean the patients can call or text me at any time?, even when I am not on duty?, and I will be obliged to response?. …I need to be sure if the government will re-imburse me for that”.	
	Quotes on perceived challenges of “Clinical decision support” recommendation	
	HW 4: “The reason I really don’t like the e-mhGAP platform is that it is not flexible at all. It tells you what to do without considering the situation. The booklet we use before is easier and much more simplified and straightforward….” HW 5: “I am not so cool with looking at my phone from time to time in front of the patient. I prefer it when the decision tree is on a banner hung on the wall in the consulting room behind he patient, I could look at it without the patient being aware I am doing that …but I can’t do that with the phone …”	

When asked specifically regarding their poor ratings for recommendations regarding “Client-to-health-provider communication,” the majority of the health workers believed emergency cases such as suicide immediately warrant face-to-face communication and were not sure they could help with digital platforms or telephone. There were specific concerns about guilt if the client’s clinical situation eventually worsened or resulted in death by suicide. Many were concerned about clinical liability in such cases, as they do not have the full picture, and some felt that it would mean working beyond their allotted time and clinical capacity without reimbursement from the authorities. Regarding the low scores on “Clinical decision support” recommendations, some of the health workers opined that the e-mhGAP is too prescriptive and would want it simplified. Many complained that checking their mobile devices in the presence of the client would affect their self-esteem.

## Discussion

Evidence-informed health systems guidelines such as the WHO-GRDI tackle health systems problems by not only framing health systems problems, but also systematically retrieving, translating and packaging the best available evidence on health systems interventions and implementation issues, and using this evidence to recommend and formulate options to solve these problems and to inform policymaking.

Using the ADAPTE method, our research team, in conjunction with the local stakeholders, successfully adapted the WHO-GRDI for mental health services in Nigeria, and we conducted a pilot implementation of the adapted WHO-GRDI. The adapted recommendations of the WHO-GRDI were found to be largely effective, acceptable and appropriate, and could be feasibly implemented for mental health services in the local setting with a focus on mobile technology. The health workers in our study perceived that digital technology would make their work easier and faster, improve their ability to relate well with other professional and specialists and expand their ranger of interventions. This is in agreement with earlier studies in LMICs that showed high level of acceptability, effectiveness and feasibility for digital interventions in terms of health workers decision support [36–38], targeted client communication [39, 40], health workers supervision and communication [41] and stock notification [42]. Our study also showed that health workers in Lagos, Nigeria, expressed some concerns and reservations regarding some recommendations of the WHO-GRDI. It is noteworthy that WHO recommends client-to-provider telemedicine only under the condition that it complements, rather than replaces, face-to-face delivery of health services and only in settings where patients’ safety, privacy, traceability, accountability and security can be measured [43]. In addition, the WHO recommends the use of digital health worker decision support only in the context of tasks that are already defined as within the scope of practice for these health workers. When implementing digital clinical decision support, it should be ensured that the device does not impact the relationship between clients and health workers negatively. It is interesting to note that at the time of writing this report, there is an ongoing feasibility trial of the electronic mhGAP-IG app for mobile devices in Nepal and Nigeria [44]. The result of the trial will further guide the implementation of the WHO-GRDI in Nigeria and other LMICs.

There are some lessons learned from our study. First, it is extremely important to have robust stakeholder engagement before, during and after the adaptation and pilot implementation of health systems guidelines. To facilitate successful implementation and cooperation of the health workers and other stakeholders, we disseminated project information early and we capitalized on the overall organizational structure of the health workers in Lagos and the MeHPriC project. We leveraged existing networks such as unions of nurses, midwives, community health workers and general practitioners in Lagos State. Second, before attempting to implement the recommendations of the WHO-GRDI, policymakers in LMICs should make sure that there is already a digital strategy in place. Also important are infrastructure (i.e. electricity, available bandwidth, mobile technology devices), legislation and policy on digital intervention, a national digital governance framework, training facilities and a plan for health workers, and existing digital health applications to support the project. Last, we believe that it was easier to integrate the WHO-GRDI recommendations within an already existing or ongoing health project. In our case, since the MeHPriC project had been up and running with some mobile telephone backup, the shift from manual to digital intervention was quite easy as all stakeholders were able to key into it.

We have prepared evidence briefs for the health managers and policymakers in Lagos State using evidence and knowledge data from this study. We structured the policy briefs to have focussed discussions and produce policy dialogues. We intend to continuously engage the stakeholders with the result of our study.

There are some limitations to our study. First, the WHO-GRDI had not been developed for mental health systems; we had to adapt and contextualize it for mental health systems in LMICs. Second, it must be noted that Lagos is the most developed state in Nigeria with a far more advanced mental health system than the remaining 35 states; thus, our results may not be generalizable to states with less developed mental health systems and rural communities. Third, our pilot implementation was short term. The 3-month duration was due to time and financial constraints. A longer-term (e.g. 6–12-month) implementation study that will access sustainability and clinical outcomes is needed. Lastly, we did not implement all the WHO-GRDI recommendations.

## Conclusions

Our study provides valuable information for those interested in adapting and implementing the WHO-GRDI and other health systems guidelines in LMICs. There is a need for further research that will embrace the views and experiences of end-users of health systems guidelines to understand and contextualize the balance between generalizability and local adaptability of health systems guidelines in LMICs.

## Data Availability

The datasets used and/or analysed during the current study are available from the corresponding author on reasonable request.

## Abbreviations

AGREE-II: Appraisal of Guidelines for Research and Evaluation
AIM: Acceptability of intervention measure
DHA: Digital Health Atlas
FGD: Focus group discussion
FIM: Feasibility of intervention measure
HS: Health systems
IAM: Intervention appropriateness measure
KII: Key informant interview
LMICs: Low- and middle-income countries
LSC: Local steering committee
MeHPriC: Mental Health in Primary Care
MH: Mental health
mhGAP: Mental Health Gap Action Program
mhGAP-IG: MhGAP intervention guide
ORIC: Organizational Readiness for Implementing Change
PHC: Primary healthcare
PIPOH: Population, Intervention, Professionals, Outcomes, and Health care settings
SF: Stakeholders’ Forum
WHO: World Health Organization
WHO-GRDI: WHO Guideline: recommendations on digital interventions for health system strengthening
